# *Marine Drugs* Best Paper Award 2015

**DOI:** 10.3390/md13021068

**Published:** 2015-02-16

**Authors:** Alejandro M. S. Mayer

**Affiliations:** *Editor-in-Chief*, Department of Pharmacology, CCOM, Midwestern University, 555 31st. Street, Downers Grove, IL 60515, USA; E-Mail: amayer@midwestern.edu

*Marine Drugs* began a “Best Paper Award” in 2013 [1,2] to recognize outstanding papers published in our journal in the area of research, development, and production of drugs from the sea, including marine natural product chemistry. We are pleased to announce the third annual “*Marine Drugs* Best Paper Award” for 2015. Nominations were selected by the Editor-in-Chief and Associate Editors of *Marine Drugs* from all papers published in 2011; reviews and articles being evaluated separately. The following five papers have won a “Best Paper Award”:

**Article Award:**
  *First Prize* 
    Gu-Ping Hu, Jie Yuan, Li Sun, Zhi-Gang She, Jue-Heng Wu, Xiu-Jian Lan, Xun Zhu, Yong-Cheng Lin * and Sheng-Ping Chen *
    Statistical Research on Marine Natural Products Based on Data Obtained between 1985 and 2008
    *Mar. Drugs* **2011**, *9*(4), 514-525; doi:10.3390/md9040514
    Available online: http://www.mdpi.com/1660-3397/9/4/514
  *Second Prize* 
    Yi Wang, Jinkai Zheng, Peipei Liu, Wei Wang and Weiming Zhu *
    Three New Compounds from *Aspergillus terreus* PT06-2 Grown in a High Salt Medium
    *Mar. Drugs* **2011**, *9*(8), 1368-1378; doi:10.3390/md9081368
    Available online: http://www.mdpi.com/1660-3397/9/8/1368
  *Third Prize* 
    Maria Mansson *, Anita Nielsen, Louise Kjærulff, Charlotte H. Gotfredsen, Matthias Wietz, Hanne Ingmer, Lone Gram and Thomas O. Larsen
    Inhibition of Virulence Gene Expression in *Staphylococcus aureus* by Novel Depsipeptides from a Marine *Photobacterium*
    *Mar. Drugs* **2011**, *9*(12), 2537-2552; doi:10.3390/md9122537
    Available online: http://www.mdpi.com/1660-3397/9/12/2537

**Review Award:**
  *First Prize* 
    Guangling Jiao, Guangli Yu *, Junzeng Zhang and H. Stephen Ewart *
    Chemical Structures and Bioactivities of Sulfated Polysaccharides from Marine Algae
    *Mar. Drugs* **2011**, *9*(2), 196-223; doi:10.3390/md9020196
    Available online: http://www.mdpi.com/1660-3397/9/2/196
  *Second Prize* 
    Sinéad Lordan, R. Paul Ross and Catherine Stanton *
    Marine Bioactives as Functional Food Ingredients: Potential to Reduce the Incidence of Chronic Diseases
    *Mar. Drugs* **2011**, *9*(6), 1056-1100; doi:10.3390/md9061056
    Available online: http://www.mdpi.com/1660-3397/9/6/1056

The Prize Awarding Committee has concluded that the five papers selected have become valuable contributions to *Marine Drugs*, as well as global marine drug discovery research, and, thus, together with the Editorial Board of *Marine Drugs*, we would like to congratulate the authors of these five articles for the significance of their scientific contributions. In recognition of their accomplishments, Dr. Sheng-Ping Chen, Dr. Weiming Zhu, and Dr. Maria Mansson will receive a prize of 600 CHF, 400 CHF, and 200 CHF, respectively, as well as the privilege to publish an additional paper free of charge in open access format in *Marine Drugs*, after the standard peer-review procedure. Dr. H. Stephen Ewart and Dr. Catherine Stanton will also be awarded the privilege of publishing an additional research paper, free of charge, in open access format in *Marine Drugs*, after the standard peer-review procedure has been completed.

*Prize Awarding Committee* 

*Editor-in-Chief* 

**Prof. Dr. Alejandro M. S. Mayer**

Department of Pharmacology, CCOM, Midwestern University, 555 31st. Street, Downers Grove, IL 60515, USA

E-Mail: amayer@midwestern.edu

*Associate Editor* 

**Prof. Dr. Jordan K. Zjawiony**

Department of BioMolecular Sciences, School of Pharmacy, University of Mississippi, MS 38677, USA

E-Mail: jordan@olemiss.edu

*Associate Editor* 

**Prof. Dr. Orazio Taglialatela-Scafati**

Dipartimento di Farmacia, Universita' di Napoli Federico II, Via Montesano 49, I-80131 Napoli, Italy

E-Mail: orazio.taglialatelascafati@unina.it

*Associate Editor* 

**Dr. Keith B. Glaser**

AbbVie, 1 North Waukegan Road, North Chicago, IL 60064, USA

E-Mail: keith.glaser@abbvie.com

*Associate Editor* 

**Dr. Peer B. Jacobson**

AbbVie, 1 North Waukegan Road, North Chicago, IL 6004-6099, USA 

E-Mail: peer.b.jacobson@abbvie.com
